# Reviews

**Published:** 1888-07

**Authors:** 


### REVIEWS.

DISSOLUTION AND EVOLUTION AND THE SCIENCE OF MEDICINE. AN ATTEMPT TO CO-ORDINATE THE NECESSARY FACTS OF PATHOLOGY AND TO ESTABLISH THE FIRST PRINCIPLES OF TREATMENT. By C. Pittfield Mitchell, M.R.C.S. London, Longmans, Green & Co. 1888.

It has long been objected to the science of Medicine that it is essentially empirical, that the facts upon which it is based have never been scientifically woven together so as to afford an unchanging groundwork of principles from which no departure can be made, no matter to what extent new facts may accumulate, nor how radical the changes may be that take place in treatment. We know that the modern practice of medicine differs *toto cœlo* from the practice of fifty years ago, and that the so-called systems of pathology over which our forefathers fought so stubbornly can scarcely be unearthed from the rubbish beneath which they lie buried. And if such was the fate that overtook the erudite systems of Boerhaave, Cullen, Broussais and Paine, it is quite within the limits of possibility that a similar consignment to oblivion is in store for our much-vaunted pathology of to-day, unless it be made to cling to some set of principles of a purely scientific character entirely exempt from change. The author of the work under consideration has made a laudable, even if a tentative, effort in this direction, and has partially succeeded in making Spencer's synthetic philosophy the basis of his scheme for giving a scientific character to practical medicine. The subject matter of Dr. Mitchell's treatise is of itself sufficiently abstract and subtle without the additional obscurity which comes of a too labored and learned style. Scientific writers, as a rule, fail to present their thoughts with that clearness and simplicity which are quite compatible with their subject, and imagine that, because they move in an atmosphere of learning, they must surround their labors with a network of abstruse and intricate phraseology. A pleasant and notable exception to this rule is Professor Tyndall, who knows how to give forth "light even from smoke," and who has invested some of the most difficult presentments of scientific work with that peculiar charm which results from a picturesque and masterly style. Herbert Spencer and Huxley likewise, unless indeed where they break down under the weight of their own thoughts and labor in a slough of misty speculation, are fond of simple Saxon and a homelike phraseology. Simplicity of this sort is not Dr. Mitchell's forte, or rather, indeed, he is apt at times to go in quest of a full and sonorous Latin derivative when the plainer Saxon, or at least more familiar form of expression, is close at hand. The term *concinnity*, for example, although it has been used by Mivart, Grant Allen and Carpenter, is not what may be termed a happy substitute for *agreement*, and is not even a well-turned derivative from its Latin original. But where there is so much matter of an important character to be considered, it is not worth while to dwell on trifling blemishes, for in comparison with the pervading value of Dr. Mitchell's work, these few peculiarities of style are no more than spots upon the sun.

In no respect, perhaps, has the far-reaching power of the doctrine of evolution to influence conditions with which it has apparently but little to do, been so strikingly illustrated as by the light its consequences have shed on the morbid states of vegetable and animal organisms. If the fruitfulness of a doctrinal system, its adaptability to a great and widely divergent variety of cases, and its elasticity under circumstances of a most diverse character, constitute a valid claim to its acceptance, then it must be conceded that the theory of evolution commends itself to man's intelligence more than any system of which the history of philosophy has preserved the record. Even a matter so remote from the dry abstractions of science as the *beautiful* has been more fully elucidated by aid of the doctrine of evolution, and both Herbert Spencer and Grant Allen have reduced to an exact scientific equivalent the fugitive data of æsthetics. Numberless other facts, likewise, which lay scattered along the highway of science, like broken ends hanging loosely from the central strand, have been gathered up by its aid and made to bear a definite and satisfactory explanation. Thus the facts of pathology which a vicious system of nomenclature, not still entirely extinct, had taught men to regard as distinct and positive entities, have been made to appear but as aberrations from natural types induced by a metamorphosis the opposite of that which marks the progress of evolution. As evolution implies a gradual ascent from simplicity of structure to complexity, from paucity of function to multiplicity through differentiation of tissue, so disease is a corresponding descent from complex structures to simpler ones, accompanied by loss of function and a return to a homogeneous substance. In a word, dissolution is a reverse process of evolution, and is subject to similar laws. It is evident then that it should be the first object of the scientific enquirer to ascertain the number and variety of those laws and their application in given cases. We strive in this manner to simplify that vast array of phenomena comprised under the term diseased conditions, and to reduce to the smallest number of distinct heads those bewildering varieties of departure from normal types. To understand fully the relation which the converse processes of evolution and dissolution hold to each other, it will be necessary that the reader have placed before him the definition of each, even though it may come clothed in the *apparently* obscure verbiage of science. We have said *apparently*, for though the terms in which Herbert Spencer has embodied his notion of evolution are harsh and uncouth, they are almost mathematically precise. Evolution, then, is "an integration of matter and concomitant dissipation of motion, during which the matter passes from an indefinite, incoherent homogeneity to a definite, coherent hetrogeneity; and during which the retained motion undergoes a parallel transformation." Thus it will be seen that evolution is a perfecting process, that it multiplies through simplicity and gives as a resultant a complexity which approaches to our highest conception of order. Dissolution, on the other hand, is "a disintegration of matter and concomitant absorption of motion, during which the matter passes from a definite, coherent hetrogeneity to an indefinite, incoherent homogeneity; and during which the retained motion undergoes a parallel transformation." Here we have a formula which represents a retrogressive metamorphosis, a degeneration of tissue accompanied by disorganization of structure and cessation of function; in a word, it represents the change from order to disorder. By an examination, therefore, of the two formulas as a comparison of their terms, it will be seen that disease in any and every shape is an infraction of the law of evolution in some of its provisions, and that it can be but imperfectly comprehended unless that aspect of the law which it violates be thoroughly understood. The first abnormal condition to which Dr. Mitchell applies his doctrine is that of inflammation. In this process there is a dispersion of corpuscles and plasma; the constituents of connective tissue occupy more space, they begin to lose their aggregated form and move away from each other gradually. We here, then, have the first step in the process of dissolution, which is disintegration, or the motion of units away from a common centre. From this statement it may be perceived that dissolution is a relative term holding a constant equation to the integration which pertains to the progress of evolution.

Inasmuch, however, as incipient dissolution is still under the control of vital force, it must be marked by some vital processes, even though they be of a retrograde character. Thus all proliferation goes rapidly forward under the influence of disintegrating forces, but as these cells are generated, instead of obeying the evolutionary tendency of hastening to a common centre, they wander aimlessly around, a certain evidence of a disorganizing tendency. The more remote consequences of inflammation, however, leave no doubt as to its dissolutional character, for no matter how rapid soever cell proliferation may be at the beginning, the inevitable termination is suppuration, sloughing or gangrene. Inseparable from this phase of inflammation, because giving rise to it, is the absorption of motion, whether thermal, chemical or mechanical. Expansion and contraction, under the influence of heat and cold, are familiar forms of this species of absorption, and the former is invariably present in inflammation. When molecular oscillation has proceeded to an advanced stage it becomes molar, and the death of large masses of inflamed tissue ensues. Thus is it proved that the terms of the definition of dissolution are verified in inflammation, and that it is a process the direct reverse of evolution.

Dr. Mitchell next considers the phenomenon of coagulation as illustrating the same general tendency of dissolution, for though some features of that phenomenon fall under the head of evolution, its general drift is towards disintegration. Indeed, these processes are so closely allied that we find them both proceeding at once in the same subject, the general tendency of either process alone determining to which class it is to be referred.

From the peculiarities of coagulation our author goes on to the consideration of neoplasms and morbid growths. These growths are the result of what may be called dissolutional cell proliferation, and are phenomena of perverted evolution tending to final dissolution. An interesting fact in connection with the formation of these growths is the comparative frequency with which they occur at points remote from the great nutritive centre—the heart. This fact is accounted for by the slowness with which they are nourished, and in a measure attest the closeness with which all sorts of retrogressive metamorphosis are connected with mal-nutritive conditions. In a similar manner Dr. Mitchell surveys the entire field of pathology, and draws from each instance of diseased conditions evidences of the truth of his theory. We have this notably illustrated in the case of pneumonia, the morbid phases of which malady display in unbroken succession all the phenomena of dissolution. Assuming that the *pneumococcus* is the provoking cause of the disorder, and the weight of authority strongly favors such a view, it is a comparatively easy matter to connect these disintegrations with the absorptions of energy as their causes.

The practical deductions which Dr. Mitchell draws from his ingenious and elaborate speculations are valuable guides to the physician in his daily relations with the sick. His philosophy points in the only true and safe direction—the ætiology of disease. It is only when the practitioner is thoroughly acquainted with the organic and environing conditions out of which diseases are born, that he can deal with them intelligently and claim beneficial results as his own. Let it be understood, however, and this is a point on which Dr. Mitchell wisely insists, that it is not necessary for these results to be positive. Indeed, it is more frequently the case that the time and inactivity of the physician is the wisest course he can pursue, and that, when he resists the temptation to meddle with nature's processes, he shows himself the true philosopher as well as the prudent physician.

Dr. Mitchell's work is a study attempted in a fruitful and salutary direction. It is an important contribution to modern medicine, and proves its author to be a thoughtful physician, a scholar of large attainments and an acute logician. C. M. O'L.

The Principles of Cancer and Tumor Formation. By W. Roger Williams, F.R.C.S. London, John Bale & Sons. 1888.

Dr. Williams remarks in his preface to this interesting volume that he had set forth his views on neoplasms, as viewed in the light of evolution, in the publications, one entitled "On the Influence of Sex in Dis ease," and the other on "Vegetable Tumors," before Dr. Pitfield Mitchell advanced his views on "Evolution and Dissolution" (*vide supra*). The author, however, frankly admits that Dr. Mitchell was not aware of these previous publications, and so stands relieved of the suspicion of plagiarism. Indeed, the coincidence in time of these two interesting monograms shows that the ideas which they represent and advocate were already in the air, and that it was only a question of time that they should receive such able expression at the hands of trained disciples of Herbert Spencer.

Dr. Williams (we believe in England a F.R.C.S. prefers the plain title of "Mr.") is an interesting writer, for the reason that he states his views in a clear and unpretending manner and wins his readers to their adoption without an effort.

The author begins the consideration of his subject by dwelling on the necessity of principles to hold the constantly accumulating mass of facts together and weaving them into a scientific fabric. The mere collation of isolated facts too often embarasses the scientific inquirer and bewilders him by their diversity. It is much more important for the man of science that he grasp the fundamental principles of his subject than that he should convert his mind into an encyclopedia of interesting information. Knowledge, to be scientific, must be systematized, and this it cannot be unless it rests upon immutable principles. It has been too much the vogue lately, in these days of microscopic, telescopic and spectrum manias, to bring together vast arrays of facts and to parade them as the latest expression of science. Dr. Williams' book is in part a protest against this tendency, and at the same time an eloquent tribute to the doctrine of evolution, which seeks to supply a scientific basis to this superabundant raw material. The chapter on growth sounds the key-note of this design by laying down a common principle to which the facts of organic and inorganic life are to be referred. In the first place, the author states, contrary to what a superficial view of vital processes would seem to favor, that life preceded organization and that function went before structure. This is eminently and significantly true, for though we usually regard function as the outcome of structure, a close observation will enable us to perceive that function is gradually determining the structure and induces modification in it. Structures, therefore, grow through function, and growth is consequently the common physiological property of all living things. But growth is not confined to living things, it is universal, applying both to organic as well as to inorganic matter, and is due to the universal tendency of like units to unite and of unlike units to separate. Crystals grow by accretion, organic matter by *intus-susception*, and these two modes of growth represent a mere accidental difference. In connection with organic growth, or that by *intus-susception*, Dr. Williams considers the modifying influences of adaptation and heredity, and prepares the reader for the results they bring about in the character and duration of tumors. The abiding quality of such modifications may be inferred from the significant words of Sir James Paget: "A mark," he says, "once made on a particle of blood or tissue, is not for years effaced from its successors." These few words speak volumes for the ineffaceability of the traces which result from heredity.

Growth, however, is but one step in the series of changes that lead to the formation of abnormal growths in the system.

The author next turns his attention to reproduction as exerting its influence in the same direction and as determining the character of the tumors that ensue from it. On the whole, it may be said that Dr. Williams' work is replete with instruction and full of suggestion to those who are interested in the varied phases of the theory of evolution.

C. M. O'L.

THE COMPARATIVE ANATOMY OF THE DOMESTICATED ANIMALS. PART I., OSTEOLOGY. By J. M. Fadyean, M.B., C.M., B. Sc. Lect. on Anat. Roy. Vet. Coll., Edinburgh. Edinburgh and London, W. & A. K. Johnston. 1888.

This very concise little treatise constitutes the first part of what proposes to be a series of similar works of the same author, treating of the anatomy of our ordinary domesticated animals. This, the osteological section of the work, is published in advance of the remainder, "in order to meet the requirements of junior students of anatomy under the recent regulations regarding the veterinary examinations." Part II., we are informed, will appear shortly.

If the one we have now in hand can be regarded in the light of foreshadowing what we may be led to expect in the others that are to follow, we can say to veterinary students that they are to be congratulated in having at their disposal so handy and valuable a work. It very fitly takes a place between the more voluminous treatise of Chauveau and the more elementary books upon the subject, and thus constitutes an admirable guide in the dissecting room as well as an examination aid.

In brief, we find, after careful review, this first part to be a neat little volume of nearly one hundred and seventy pages, illustrated by some 132 wood cuts, figuring all the important bones of the various domesticated animals which have been so ably treated, as to their osteology, in its pages. Very properly, it first points out, as an introduction, the classificatory position of the domesticated animals in the system; when next follows a very excellent section devoted to the leading features of bones and their growth generally. Both of these chapters are amply illustrated by characteristic figures. Next comes a full consideration of the skeleton, the horse being chosen as sort of an example, and the skeletons of the cow, pig, sheep, dog and cat compared with it throughout the work, with the differences and similarities consistently pointed out. The type is clear and luminous, with a very useful advantage being taken of using larger type for salient features treated of in any particular paragraph.

Occasionally we meet with a little slip, as for instance, in describing the *tarso-metatarsus* of the fowl, the author is made to say that " no free tarsal bones" are to be found in its skeleton, whereas, further along in the same paragraph we are informed that "the first or innermost metatarsal is rudimentary, and united by ligament, * * * * " in which case it is most assuredly "free," whether it be rudimentary or otherwise (p. 166). In short, as we all know, the hallux metatarsal *is free* in the fowl. But such evident oversights can be easily remedied in a succeeding edition; and, too, we would recommend that should this excellent little manual go to another edition, an improvement be made in some of the figures, as for example, the figure of the "Manus of Dog," on page 136, which is not only roughly engraved, but in detail quite indistinct; and this applies to a number of others, although on the whole, notwithstanding the fact that the illustrations are somewhat coarsely executed, they are bold, accurate and helpful. Further, we would suggest that a complete index at the end of the volume would be an improvement and of material assistance.

Beyond these few points of criticism, however, we have nothing but praise left for this truly welcome work, and it quite fully meets all that it pretends to be, a useful handbook for the student of veterinary anatomy, who can ill afford to be without a copy, while the succeeding Parts will be looked for by all veterinarians with interest. R. W. S.

IV.—A Treatise on the Diseases of the Dog, being a Manual of Canine Pathology Especially Adapted for the Use of Veterinary Practitioners and Students. By John Henry Steel, M.R.C.V.S., A.V.D. London. Longmans, Green & Co. 1888.

The American reader will congratulate himself and the veterinary profession on receiving this charming and instructive book, of 287 pages, good paper and with clear type. The American medical book publisher would do well to take a few lessons from Longman, Green & Co. Professor Steel's previous works are already so well known that a new book from his pen will find a ready welcome, but the study of this book will prove a greater pleasure than is expected.

Chapter I., the introduction, refers to what has already been done in canine pathology; gives the general differential characteristics of the canines from other species, of families of these from others, and of the individual from his own breed. There is given a general outline of the general symptoms in dogs, the pulse, respiration, visible mucous membranes, general behavior, secretions and temperature. Professor Steel dwells upon the peculiar nervous system of the dog in its influence on the course, prognosis and treatment of his ills, and as necessitating gentleness on the part of the attendant. His sentence in prognosis, "but we must always remember the liability of dogs to sudden death from convulsions," will be a useful authority for the practitioner to quote in the many unfortunate cases which terminate in that trouble. Professor Steel makes an unfortunate recognition, without disapproval, of the vicious system in most English speaking schools of separating the veterinarian from the canine practitioner—a distinction that is not made on the continent and in some English speaking schools.

Chapter II. is an excellent article on canine pharmacy, Materia Medica and therapeutics; it is subdivided into the various forms in which remedies can be administered, and then into classification, according to the action of the drugs. To this is appended a few pages of external applications and minor surgery.

The next nine chapters are devoted respectively to the diseases of each system; the blood, the circulatory system, the respiratory system, disorders of the digestive apparatus, the urinary apparatus, the generative apparatus, the nervous system, the organs of special sense and the locomotor system. Each of these is headed by a review of the anatomy of the part and to each is appended an article on the surgery required in the treatment of any of the troubles of the organs.

Chapter XII. is devoted to poisoning and the antidotes.

Chapter XIII. treats of tumors, wounds, etc., and other treatment.

In termination is appended a "Tabular Statement of Doses and Special Action of Medicines in Canine Practice."

To Professor Steel we owe one of the richest additions to veterinary literature that we have had for some years; the book is well modeled and classified; it is susceptible of enlargement, but will remain the foundation of future canine pathology.

